# Contextual Modulation of Feedforward Inputs to Primary Visual Cortex

**DOI:** 10.3389/fnsys.2022.818633

**Published:** 2022-02-01

**Authors:** Benjamin S. Lankow, W. Martin Usrey

**Affiliations:** Center for Neuroscience, University of California, Davis, Davis, CA, United States

**Keywords:** LGN, TRN, vision, thalamus, feedback

## Abstract

Throughout the brain, parallel processing streams compose the building blocks of complex neural functions. One of the most salient models for studying the functional specialization of parallel visual streams in the primate brain is the lateral geniculate nucleus (LGN) of the dorsal thalamus, through which the parvocellular and magnocellular channels, On-center and Off-center channels, and ipsilateral and contralateral eye channels are maintained and provide the foundation for cortical processing. We examined three aspects of neural processing in these streams: (1) the relationship between extraclassical surround suppression, a widespread visual computation thought to represent a canonical neural computation, and the parallel channels of the LGN; (2) the magnitude of binocular interaction in the parallel streams; and (3) the magnitude of suppression elicited by perceptual competition (binocular rivalry) in each stream. Our results show that surround suppression is almost exclusive to Off channel cells; further, we found evidence for two different components of monocular surround suppression—an early-stage suppression exhibited by all magnocellular cells, and a late-stage suppression exhibited only by Off cells in both the parvocellular and magnocellular pathways. This finding indicates that stream-specific circuits contribute to surround suppression in the primate LGN and suggests a distinct role for suppression in the Off channel to the cortex. We also examined the responses of LGN neurons in alert macaque monkeys to determine whether neurons that supply the cortex with visual information are influenced by stimulation of both eyes. Our results demonstrate that LGN neurons are not influenced by stimulation of the non-dominant eye. This was the case when dichoptic stimuli were presented to classical receptive fields of neurons, extraclassical receptive fields of neurons, and when stimuli were appropriate to produce the perception of binocular rivalry.

## Introduction

Throughout the visual system, information is carried in parallel processing streams that contribute to functionally specific computations. The lateral geniculate nucleus (LGN) of the thalamus is remarkable in that the feedforward inputs of many parallel streams originating in the retinas (parvocellular, magnocellular, and koniocellular channels; ipsilateral and contralateral eye channels) are kept anatomically segregated at a cellular level within the structure, making functional characterization of individual streams more approachable for the physiologist than in later stages of the visual system. Opportunities for stream mixing exist through local interneurons, thalamic reticular nucleus (TRN), and cortical feedback (among others), which opens the interesting possibility that circuitry subserving inter-stream interactions, if present, could be differentiated based on the time course of the interactions. The LGN thus provides an excellent model for understanding the processing characteristics inherent to the individual streams, and for understanding the extent and mechanism of stream mixing in the earliest stages of visual processing.

For many neurons in the LGN, stimuli that extend beyond the classical receptive field of the neuron have a suppressive effect on the cells’ spiking activity (Hubel and Wiesel, 1961; Murphy and Sillito, 1987; Solomon et al., 2002; Bonin et al., 2005; Alitto and Usrey, 2008; Camp et al., 2009; Archer et al., 2021). This process is thought to represent a contrast-dependent gain control mechanism (Bonin et al., 2005), and recent evidence has suggested that there are stream-dependent differences in the magnitude of extraclassical suppression, with extraclassical suppression being stronger in the magnocellular stream than in the parvocellular stream (Solomon et al., 2002; Webb et al., 2002, 2005; Alitto and Usrey, 2008; Archer et al., 2021), and that within the magnocellular stream, extraclassical suppression is stronger in Off-cells than in On-cells (Archer et al., 2021). It remains unclear whether the temporal dynamics of suppression in these streams are similar, and whether such Off-On dichotomy exists within the parvocellular stream.

Such ambiguity is also evident in the characterization of interactions between parallel streams in the visual system originating from the two eyes. Even though individual cells in the magnocellular and parvocellular layers of the LGN only receive direct input from one retina (Guillery, 1971; also noted by De Valois et al., 1958), the potential for binocular mixing exists through interlaminar connectivity, feedback from the thalamic reticular nucleus (TRN), and feedback from V1. While it is generally agreed that cells in the LGN of the carnivore exhibit moderate binocular interaction, the question of whether signals from the two eyes interact in the LGN of the primate is less clear; roughly half of all published studies that have investigated binocular modulation of neuronal activity in the primate LGN have reported its occurrence, but the magnitude, stream specificity, and incidence are unclear (reviewed in Howard, 2012). Schroeder et al. (1990) reported that almost all of their recording sites in the primate LGN showed some form of binocular modulation; other groups (Marrocco and McClurkin, 1979; Rodieck and Dreher, 1979; Dougherty et al., 2021) have also found instances of binocular modulation in the LGN, although the results are heterogeneous. Still, other groups have found no evidence of such interaction (e.g., Lehky and Maunsell, 1996). A similar controversy regarding binocular interaction in the LGN centers on the role of the LGN in binocular rivalry. While fMRI studies have shown extremely strong eye-specific modulation of the BOLD response in the LGN correlating with perceptual suppression during binocular rivalry (Haynes et al., 2005; Wunderlich et al., 2005), single-unit studies have not reported any evidence of perceptual modulation in the LGN (Lehky and Maunsell, 1996; Wilke et al., 2009). Moreover, as the LGN comprises the most experimentally tractable arrangement of parallel visual pathways in the early visual system, such inconsistencies have left unclear whether binocular operations are reflected within distinct visual pathways.

The primary goals guiding the experiments (all conducted with alert macaque monkeys) described in this study were: (1) to determine the characteristics of surround suppression in the parallel streams of the LGN, (2) to determine the extent to which non-dominant eye stimulation influences the spiking activity of LGN cells, and (3) to determine the magnitude of the effect of perceptual suppression on the sensitivity of LGN cells. In our investigation of the stream-specificity of monocular surround suppression in the LGN, we found that surround suppression is carried predominantly by Off-channel cells, with magnocellular and parvocellular Off cells showing distinct temporal dynamics, indicating a functional specialization for these cells. Based on previous studies, we had the advantage of knowing that any binocular effects, if present, were likely to be small. We, therefore, designed our experiments examining interocular interactions with a focus on maximal sensitivity and statistical power. Our results show that binocular signal interactions are not evident in either the parvocellular or magnocellular layers of the primate LGN. These results demonstrate that in the earliest stages of visual processing, monocular visual streams within the magnocellular and parvocellular pathways are kept strictly independent within these layers.

## Materials and Methods

### General Procedures

Two female macaque monkeys (*Macaca mulatta*) were used in this study. All procedures were approved by the Institutional Animal Care and Use Committee at the University of California, Davis, and conformed to NIH guidelines. Surgical procedures have been described previously (Briggs and Usrey, 2009). Briefly, under full surgical anesthesia, a head post and a recording cylinder positioned above the LGN were secured to the skull. Single-unit recordings (*n* = 127) were made from the LGN in the alert animals using platinum-in-glass electrodes (1 MΩ; Alpha Omega). Voltage recordings were amplified and recorded by a PC equipped with a Power 1401 data acquisition system and Spike2 software package (Cambridge Electronic Design).

### Viewing Apparatus

Stimuli were presented on two gamma-calibrated Sony Trinitron GCM-F520 monitors running at 100 Hz with a resolution of 944 × 708 pixels. The viewing distance was 61 cm. The stimuli were generated on two synchronized VSG2/5 visual stimulus generators (Cambridge Research Systems, Rochester, England). The mean luminance of each monitor was 38 candelas/m^2^. Animals were trained to fixate on a central dot viewed through a custom-built Wheatstone-style stereoscope; eye position was monitored with an infrared video eye tracker (Applied Science Laboratories; refresh rate 240 Hz). The stereoscope was aligned and vergence and accommodation were matched by replacing the mirrors with beamsplitters and aligning a central fixation point on each monitor to an LED located an equal distance behind the beamsplitters. Once the stereoscope was aligned, the beamsplitters were replaced with mirrors. For all experiments, identical high-contrast annular checkerboards surrounded the fixation points in order to stabilize binocular fusion.

### Establishing Cell Types in the Alert Monkey

**Figure 1:**
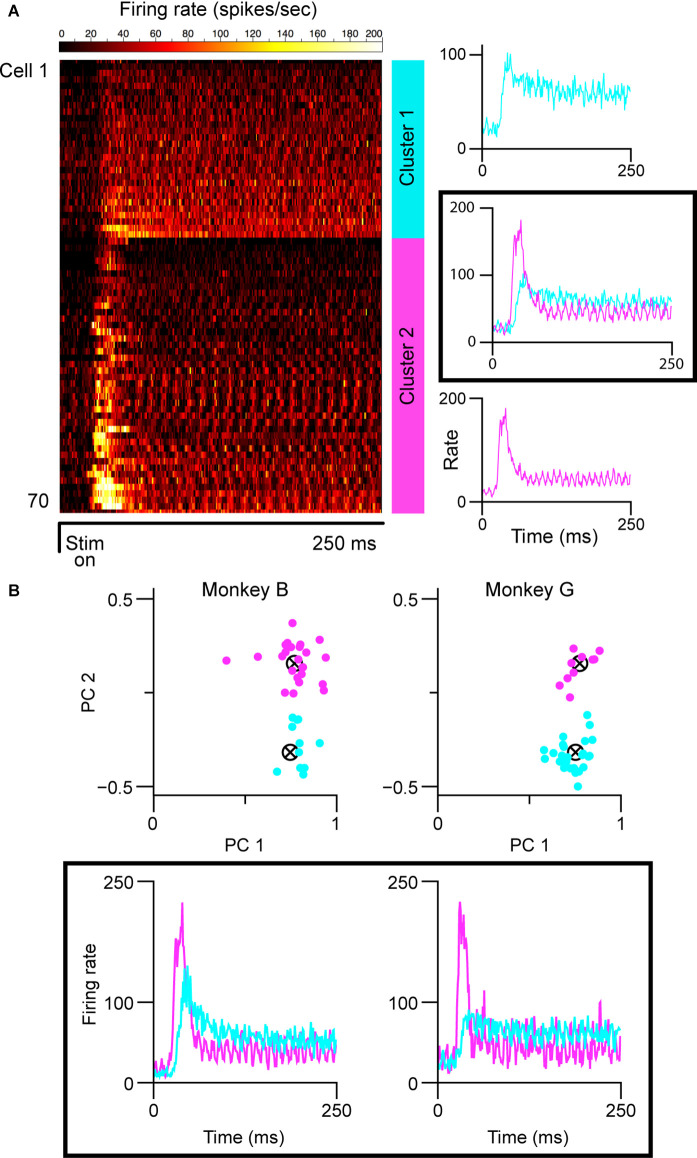
Cell partitioning based on spiking responses elicited by static gratings. **(A)** Results of AP algorithm. Mean spiking responses of all cells in this experiment are shown on the left (*n* = 71). Two clusters were found (mean group firing patterns shown at right): one with sustained spiking responses, longer latencies, and lower peak firing rates, and one with transient spiking patterns, shorter latencies, and higher peak firing rates. The “ripples” in the firing patterns of the transient group are caused by the cells responding to the monitor refresh (100 Hz). **(B)** Results of k-means clustering. Clustering run separately for each monkey shown at top; scatterplot shows the projection of normalized responses onto the first two principal components of the respective data matrices. Clustering was seeded with two centroids. Mean spiking response patterns from each group are shown at the bottom. In all panels, putative magnocellular neurons indicated in magenta, putative parvocellular neurons indicated in blue.

Both the lack of histological verification of recording sites and complicated layering in the caudal/parafoveal region of the LGN introduce a certain level of subjectivity in reporting the magnocellular/parvocellular classification of recorded neurons. We, therefore, partitioned cell classes based on spiking response patterns using two clustering algorithms. For characterization, we used each cell’s averaged peri-stimulus time-histogram (PSTH) based on responses to optimally-sized static gratings (Figure 1A). We then ran the affinity propagation (AP) algorithm (Frey and Dueck, 2007), which identified two clusters within the collection of response data. We next verified the groupings with k-means (KM) clustering seeded with two centroids. The input to the AP algorithm was a similarity matrix composed of the negative squared errors between each cell’s PSTH. KM clustering was performed on the projections of the normalized PSTHs onto the first two principal components of the PSTH data matrix (Figure 1B). Both algorithms converged on one cluster with short response latencies, transient firing profiles, and high peak firing rates, and another cluster with longer response latencies, sustained firing profiles, and lower peak firing rates, corresponding to putative magnocellular (M) and parvocellular (P) neurons, respectively. KM included seven more cells in the sustained group (34 vs. 28) than affinity propagation did. In cases where the recording layer was unambiguous (electrode penetration traversed all six layers of the LGN, verified by expected transitions in eye dominance and associated shifts in observed response properties), parvocellular/magnocellular classification matched the KM clustering results, but several parvocellular cells were labeled as “transient” by the AP algorithm. The partitioning method used did not change the conclusions drawn from the experiments. The figures in this article were generated from the K-means groupings.

### Measuring Responses to Monocular and Binocular Flashed Gratings

Our first experiment characterized the spiking responses elicited by LGN cells to brief presentations of static sine-wave gratings. We used a range of stimuli designed to measure monocular surround suppression, binocular interactions between congruent stimuli, and interocular transfer of surround suppression. For every cell, the location of the receptive field center was mapped by hand, after which an area-summation tuning curve was generated using drifting gratings in order to determine the cell’s preferred stimulus size. The preferred phase was determined from presentations of static gratings centered over a cell’s receptive field. Spatial frequency was selected based on cell class and eccentricity and confirmed with spatial frequency tuning curves for a subset of cells. This was important to minimize suppressive influences from the classical surround on our measurements of extraclassical suppression (Alitto and Usrey, 2008). Because our area summation tuning curves always indicated an optimal stimulus size of diameter greater than a half-cycle of the stimulus spatial frequency, it is unlikely that our choices of spatial frequencies drove significant suppressive effects from the classical receptive field.

Animals fixated on a binocularly-presented central fixation point; after 250 ms, one of six pseudo-randomized stimulus combinations was presented for 250 ms. The stimulus set (*n* = 6 stimuli) consisted of monocular or binocular optimally-sized gratings, monocular or binocular large (4–8 degree) gratings, and an optimal grating presented to the dominant eye simultaneously with a small or large surround annulus presented to the non-dominant eye. The inner diameter of the annuli was equal to the outer diameter of the grating presented to the dominant eye. The phase of the gratings was always matched in the two eyes. Spikes were binned at 1 ms resolution and averaged over all trials within conditions. Temporal comparisons between conditions were made using the difference in the cumulative spike counts elicited by the stimuli. Reported measurements of response latency and suppression latency are the x-intercepts of linear fits to the baseline-subtracted initial stimulus response and the cumulative suppression curves, respectively.

### Disparity Tuning Measurements

In 36 of the cells, we calculated tuning to binocular disparity in order to determine whether there is an influence of local phase or positional differences on binocular modulation in the LGN. For all cells, we used drifting gratings with a diameter of 1 degree, temporal frequency of 4 Hz, and spatial frequency that elicited a vigorous response (typically 1.5 cycles per degree, not less than 1 cycle per degree). The stimulus (oriented 90 degrees) in the dominant eye was held fixed over the cell’s receptive field location, and the horizontal position of the stimulus in the non-dominant eye (of equal size, spatial frequency, temporal frequency, and contrast) was varied pseudorandomly across trials for several repeats of nine positional steps to include crossed phase match, zero disparity, and uncrossed phase match. The significance of modulation was assessed with an ANOVA, and we quantified disparity tuning using a disparity discrimination index (Cumming and Deangelis, 2001; Watanabe et al., 2002):


$$DDI=\frac{R_{\max}-R_{\min}}{R_{\max}-R_{\min}+2RMSE}$$

where *R_max_* and *R_min_* are the maximum and minimum averaged rates and RMSE is the residual variance around the mean rates of all disparities. We analyzed both the mean and F1 responses of the cells; the results were not qualitatively different and we report the results of the F1 analysis here.

### Reverse-Correlation Analysis

We used a reverse-correlation procedure to estimate the temporal kernels of LGN cells during monocular viewing and binocular rivalry. A depiction of the method is shown in Figure 7. During 2.1-s binocular fixation intervals, a 1-degree grating with a spatial frequency of 1.5 cycles/degree was presented monocularly in an isolated cell’s receptive field. The phase of the grating (0 degrees or 180 degrees) was updated every 10 ms according to a random sequence that was used for all cells and stimulus conditions. The stimulus sequence was a 12,000-frame binary noise sequence, presented in 2.1-s trial epochs that overlapped by 100 ms; therefore, one full cycle of the stimulus sequence required 60 2.1-s trials. The full trial sequence and evoked spikes were “stitched” back together to form a complete stimulus-response set and generate temporal kernels, computed as:


$$D\;(\tau )=\frac{\sum_{i\;=\;1}^{n}s\;(t_{i}-\tau )}{T\sigma_{s}^{2}}$$

where *n* is the total number of spikes, *t_i_* are the spike times, τ is the time before the spike, T is total stimulus duration, and $\sigma_{s}^{2}$ is the variance parameter of the stimulus (see Dayan and Abbott, 2001). Comparisons between conditions were based on the magnitude (*L_2_* norm) of the temporal kernels.

**Figure 2:**
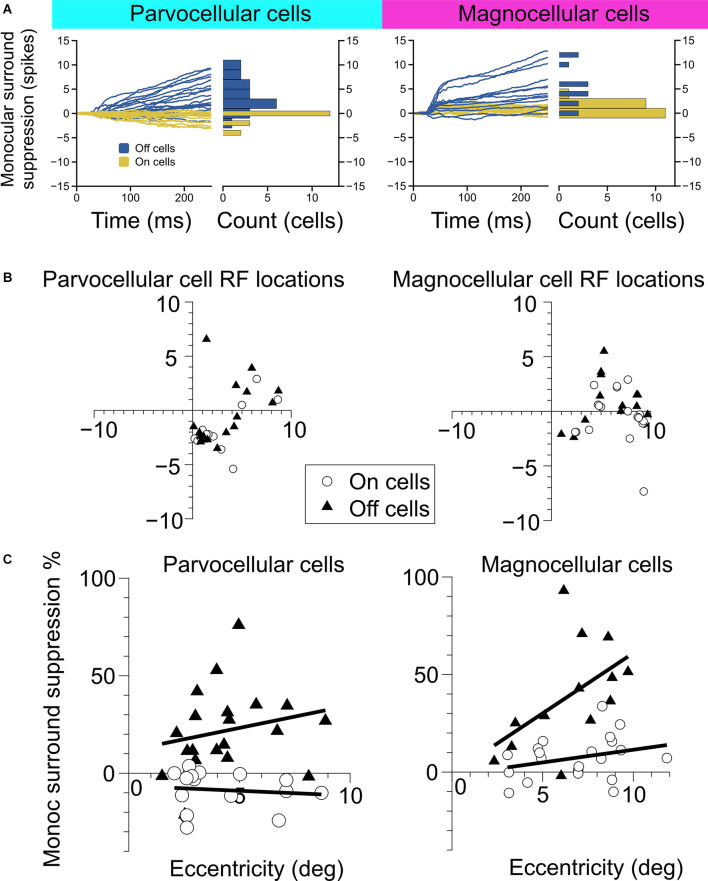
Measurement of monocular surround suppression in On-cells and Off-cells of the parvocellular and magnocellular pathways. **(A)** The cumulative difference between spikes elicited by an optimally-sized monocular grating and spikes elicited by a large monocular grating. Histograms show the total difference in the number of spikes elicited by the stimuli. Off cells displayed statistically greater monocular surround suppression than On cells in both the parvocellular and magnocellular groupings. **(B)** Receptive field location of cell recordings in this experiment. **(C)** Surround suppression as a function of receptive field eccentricity in parvocellular and magnocellular cells. In both cell groups, Off cells showed greater monocular surround suppression than On cells. Bold lines depict linear fits to On cell and Off cell surround suppression in relation to receptive field eccentricity.

**Figure 3:**
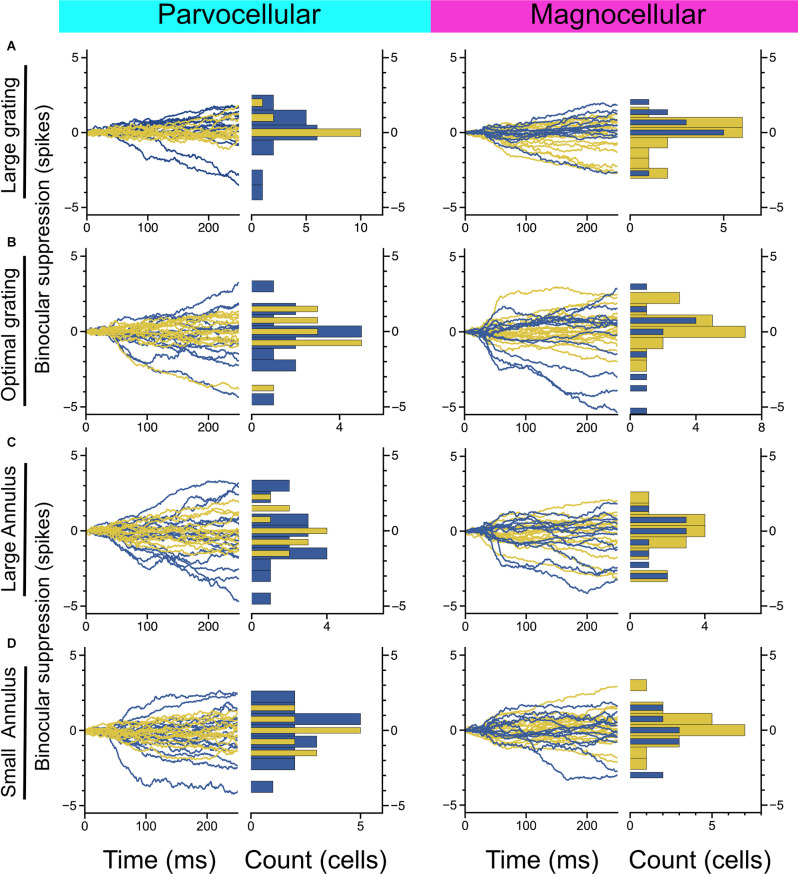
Measurement of binocular interaction in On-cells and Off-cells of the parvocellular and magnocellular pathways. **(A)** Cumulative differences in spiking elicited by a large-sized probe grating presented to the dominant eye alone vs. with a large grating presented to the non-dominant eye. **(B)** Cumulative differences in spiking elicited by an optimally-sized probe grating presented to the dominant eye alone vs. with an equally-sized grating presented to the non-dominant eye. **(C,D)** Cumulative differences in spiking elicited by an optimally-sized probe grating presented to the dominant eye alone vs. with a large annulus **(C)** or small annulus **(D)** presented to the non-dominant eye. Stimulus configuration is stated to the left of plots.

**Figure 4:**
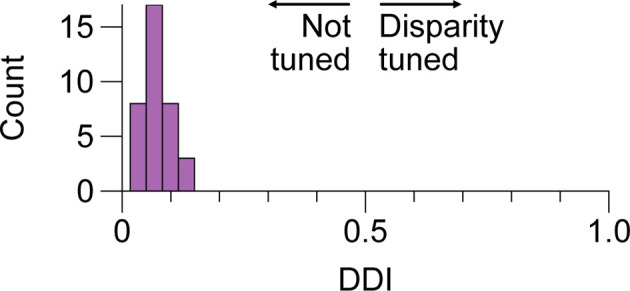
No tuning for positional disparity in LGN cells of the M and P layers. Distribution of disparity discrimination indices (DDI) for a sample of 36 LGN cells included in this study. Higher values indicate more tuning. We did not observe evidence of modulation by disparity in any of the cells; P and M cell DDIs were not distributed differently and are aggregated here. LGN, lateral geniculatenucleus.

**Figure 5:**
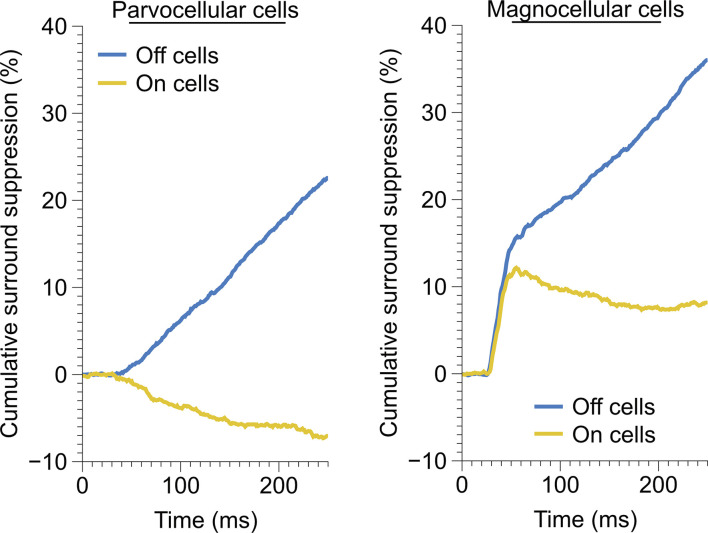
Time-course of monocular surround suppression in the On and Off cells of the parvocellular and magnocellular groupings. Left, time-course of monocular surround suppression in parvocellular cells (Off, *n* = 20 cells; On, *n* = 17 cells). Only Off-channel parvocellular cells showed surround suppression, On-channel parvocellular cells show mild surround facilitation. Right, time-course of monocular surround suppression in magnocellular cells (Off, *n* = 13 cells; On, *n* = 21 cells). Both channels exhibit fast suppression; late suppression is only present in the Off channel.

**Figure 6:**
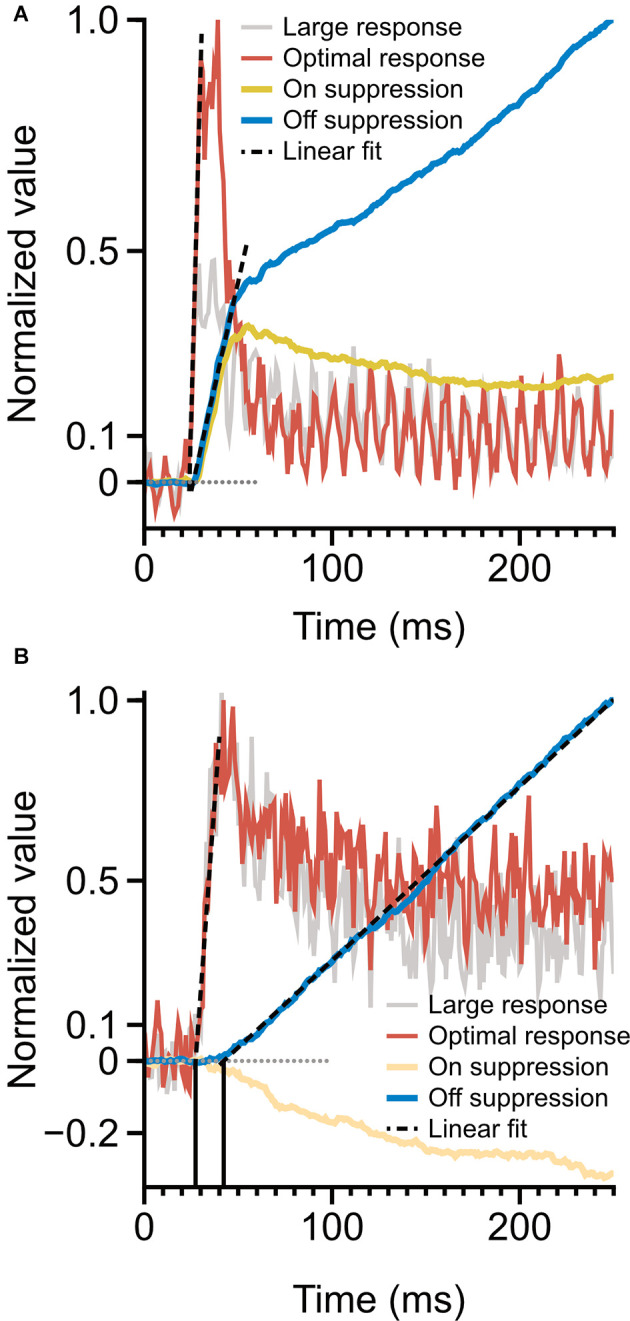
Time-course of spiking response and monocular surround suppression. **(A)** Magnocellular cells, monocular surround suppression in the On and Off channels overlaid on mean magnocellular-cell spiking response. Late-stage suppression becomes active as the rate transient drops to plateau. The onset of suppression is simultaneous with spiking response onset. **(B)** Parvocellular cells, monocular surround suppression in the On and Off cells overlaid on mean parvocellular-cell spiking response. Dotted lines depict linear fits to response onset and to the rising portion of cumulative suppression. The onset of suppression is delayed by 15 ms (vertical lines) relative to spiking response. Latencies are computed as the x-intercept of linear fits.

**Figure 7:**
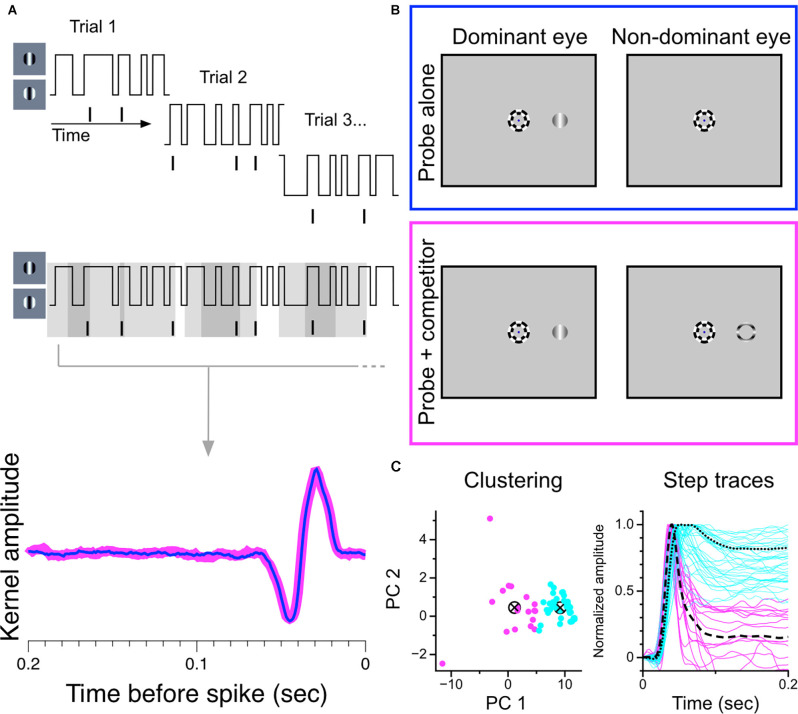
Construction and partitioning of temporal kernels recovered during monocular stimulation and during binocular rivalry. **(A)** Construction of temporal kernels. A grating centered over a cell’s receptive field updates its phase every 10 ms according to a random binary noise sequence. Overlapping segments of the stimulus are presented in 2.1 s trials. Temporal kernels are generated from the combined stimulus-response set. **(B)** Stimulus configuration. Trials were randomly interleaved in which either the monocular probe grating was presented alone to the dominant eye or was presented simultaneously with a suppressor stimulus in the other eye to generate binocular rivalry. The full noise sequence was completed for both trial conditions. Suppressor could be a thin annulus (shown here) of an orthogonally-drifting grating, or a full drifting grating, orthogonal to probe. **(C)** Partitioning of temporal kernels. Normalized data were projected onto the first two principal components of the data matrix and clustered using k-means seeded with two centroids. Partitioned step responses (normalized) are shown at right.

**Figure 8:**
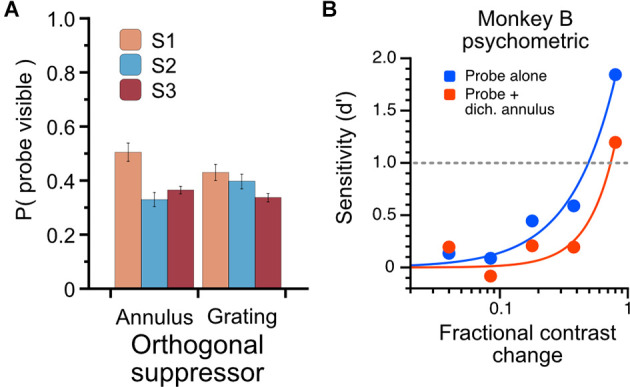
Characterization of psychophysical suppression elicited by dichoptic probe-annulus pairs. Three human subjects each viewed the modulating probe grating and dichoptic suppressor for a total of six full runs through the noise sequence; subjects indicated with a button press when the probe was visible. **(A)** The total proportion of stimulus duration in which probes were dominant. Dichoptic annuli and dichoptic gratings drove similar perceptual suppression for subjects (mean proportion of probe visibility: annulus suppressor, 0.4 ± 0.09; grating suppressor, 0.39 ± 0.05). **(B)** Psychophysical influence of dichoptic annulus on contrast sensitivity in Monkey B. Contrast change-detection performance was measured using a 2-alternative forced-choice task for probe-alone condition (blue) and probe plus dichoptic annulus (red). Contrast sensitivity was substantially lower when a thin annulus was presented to the non-dominant eye simultaneously with the probe stimulus.

Monocular and dichoptic stimulus presentations were randomly interleaved. During dichoptic trials, a thin annulus (inner diameter = 1 degree, outer diameter = 1.1–1.2 degrees) of a drifting grating (drifting orthogonal to probe orientation, temporal frequency of 4 Hz) was presented in the corresponding location of the non-dominant eye. This configuration drives robust binocular rivalry between the probe and the annulus (Figure 8), and was intended to avoid direct confounds of (spatial) binocular interaction not associated with perceptual competition. In a subset of cells (*n* = 14), a high-contrast grating drifting orthogonally to the probe was used instead of an annulus; there was no detectable difference in these results, and the data from the two suppressor conditions were aggregated.

### Characterization of Perceptual Suppression Elicited by the Stimulus

In three human observers (all male, two naïve, one author), we measured the temporal properties of perceptual suppression elicited by the reverse-correlation stimulus set and the total proportion of probe visibility during all trials (Figure 8). Observers viewed the reverse-correlation stimulus set through a stereoscope on the same experimental rig used in the monkey experiments and were instructed to press a button when the probe was visible; data shown are from responses averaged over six full runs (6 × 60 2.1-s trials, runs conducted on separate days) of the stimulus sequence. In our human observers, the probes were reported visible less than half of the time (mean proportion visible = 0.40, SD = 0.09). To confirm that the probe-annulus stimulus configuration elicited a similar effect in the monkeys, we designed a psychophysical experiment that avoided the ambiguous perceptual report by using a contrast change-detection paradigm. One monkey was trained to fixate a central dot and attend to probe stimuli (drifting gratings, 50% contrast) presented to the left and right of the fixation point in one eye (two gratings, mirrored eccentricities of 3–8 degrees); on half of the trials, a thin annulus drifting orthogonally to the probe was simultaneously presented (dichoptically) to the other eye to induce binocular rivalry. After 0.5 s, a pseudoramly-drawn contrast increment was added to one of the probes for 0.5 s, after which the monkey indicated which of the probes had changed by making a saccade to one of two lateral targets. We measured sensitivity in each contrast-change magnitude as *d’* = *Z* (*hits*) − *Z* (*false alarms*) (Macmillan and Creelman, 2004).

### Eye-Position Control Analyses

We also used a *post-hoc* analysis of eye position during trials in order to identify and eliminate trials or recordings in which differences between the eye positions across different stimulus conditions may have affected our results. For each trial, we measured the average Mahalanobis distance (Mahalanobis, 1936) between the measured [x, y] eye positions over that trial and the distribution of eye positions calculated from all trials in the appropriate comparison condition. For a trial with N discrete eye position measurements, the average distance is given by:


$$\frac{1}{N}\sum_{i\;=\;1}^{N}\sqrt{{\left({\vec{x}}_{\iota }-\vec{y}\right)}^{T}Q\;({\vec{x}}_{\iota }-\vec{y})}$$

Where each ${\vec{x}}_{i}$ is an [x, y] eye position measurement, $\vec{y}$ is the mean [x, y] eye position of the comparison condition, and Q is the 2 × 2 inverse covariance matrix of the comparison measurements. We excluded trials with an average Mahalanobis distance greater than 2 and excluded recordings from a comparison condition in which 20% or more of the trials exceeded this threshold. Approximately 15% of traces were excluded in each comparison condition based on this threshold. For the reverse-correlation study, we did not eliminate single trials in order to preserve the full sequences used to generate the temporal kernels in the two different trial conditions. In that study, three recordings were elided from our analysis that showed large average distances from the probe-only distributions in the binocular rivalry condition.

## Results

### Distinguishing LGN Cell Types in the Alert Macaque Monkey

We partitioned our sample of cells into putative parvocellular (sustained) and putative magnocellular (transient) cells based on recorded responses to 250 ms presentations of optimally-sized static gratings (Figure 1A; see “Materials and Methods” section). To ensure that our results were not affected by our choice of the clustering algorithm, we employed two widely-used methods. The affinity propagation (AP) algorithm, which does not require that the number of clusters be specified, was used first; a second set of analyses was then run using the results of k-means (KM) clustering. As shown in Figure 1B, both the AP and KM algorithms converged to one group of cells with short visual latencies, transient firing patterns, and high firing rates (putative magnocellular neurons), and one group of cells with longer visual latencies, sustained firing patterns, and lower firing rates (putative parvocellular neurons). Throughout the rest of this article, we refer to these partitioned classes as magnocellular and parvocellular neurons.

### Monocular and Binocular Surround Suppression in the LGN

We examined the magnitude and time-course of monocular surround suppression in the two groups of cells, additionally partitioned into On-center vs. Off-center cell types, by comparing responses to preferred-size monocular gratings with responses to large monocular gratings (static gratings were used in this experiment and were presented at the cell’s preferred phase). The traces in Figure 2A show the difference in the total number of elicited spikes between the conditions as a function of time for each cell in the group (positive values indicate response suppression, negative values indicate response facilitation). We observed statistically significant surround suppression both in the parvocellular cells and the magnocellular cells. Surprisingly, among the parvocellular cells, only Off cells exhibited surround suppression (*t*-test, *p* = 0.0001). Among the magnocellular cells, we observed statistically significant surround suppression in Off cells (*t*-test, *p* = 0.0006) and On cells (*t*-test, *p* = 0.003). Magnocellular Off cells exhibited far greater surround suppression than magnocellular On cells (Welch’s *t-test*, *p* = 0.0028). Magnocellular Off cells did not show stronger surround suppression than parvocellular Off cells (Welch’s *t*-test, *p* = 0.25), but there was a significant difference in the strength of magnocellular On-cell suppression and parvocellular On-cell suppression (Welch’s *t*-test, *p* < 0.0001). These differences in suppression were not due to sampling biases in receptive field eccentricities (Figures 2B,C). For cells in which surround suppression was present, suppression generally increased with eccentricity, although there was more influence of eccentricity within Off cells than On cells (Figure 2C).

We next considered whether there is evidence of binocular modulation in any of the cell groups. The rows of Figure 3 show comparisons of responses to monocular and binocular large (4–8 degree) gratings (Figure 3A), monocular or binocular optimally-sized gratings (Figure 3B), an optimal grating presented to the dominant eye alone or simultaneously with a large annulus presented to the non-dominant eye (Figure 3C), or an optimal grating presented to the dominant eye alone or simultaneously with small surround annulus presented to the non-dominant eye (Figure 3D). After mitigating eye position differences between the monocular and binocular conditions (see “Materials and Methods” section), we did not observe statistically significant binocular modulation in any cell group or trial condition. To be able to rule out a dependency of binocular modulation in the LGN on the stimulus phases in the two eyes, we also generated disparity tuning curves for 36 of the cells in this study. Although Xue et al. (1987) investigated phase-disparity tuning in the LGN of cats using full-field gratings, we are not aware of any studies that have reported measurements of positional disparity tuning in the LGN of alert monkeys. We manipulated disparity by presenting drifting gratings centered on cells’ receptive fields in the dominant eye while varying the horizontal position of a drifting grating of equal spatial frequency, temporal frequency, size, and contrast in the non-dominant eye (see “Materials and Methods” section). ANOVA analyses did not find evidence of interactions approaching statistical significance in any of the 36 cells; we computed a disparity discrimination index (DDI) for each cell and found no evidence for disparity modulation (median DDI = 0.07) in the sample (Figure 4).

We observed drastically different temporal characteristics in surround suppression exhibited by parvocellular and magnocellular cells. The mean time-course of suppression in Off cells and On cells in these groups is plotted in Figure 5. Note that the slope of the cumulative suppression traces is proportional to the strength of suppression. Magnocellular Off cells exhibit a very strong early phase of suppression followed by a weaker late-stage suppression; Magnocellular On cells exhibit a similar early-stage suppression as magnocellular Off cells, but no late-stage suppression (in fact, there is a mild late-stage facilitation, or possibly release from suppression). Parvocellular cells do not exhibit early-stage suppression, but parvocellular Off cells exhibit late-stage suppression of almost identical strength to that of magnocellular Off cells. Parvocellular On cells, conversely, show mild late-stage facilitation, similar to magnocellular On cells.

A comparison of the timing of firing-rate responses and the time-course of surround suppression is illustrated in Figure 6. Magnocellular cells (Figure 6A) show a clear demarcation in early-stage and late-stage surround suppression. During the initial response transient, both On cells and Off cells show strong surround suppression; as the response transient drops to a plateau, surround suppression in the On cells and Off cells diverges. This figure suggests a shared early-stage suppressive mechanism, but also suggests that the late-stage suppression may be specific to the Off channel. The onset of surround suppression in parvocellular Off cells is delayed relative to parvocellular cells’ response latency (Figure 6B). The close similarities between late-stage suppression in the magnocellular and parvocellular cells suggest that there is a shared late-stage suppression pathway common to the entire Off channel.

### Influence of Perceptual Suppression on LGN Cell Response Properties

We used a reverse-correlation procedure to estimate the temporal kernels of LGN cells during monocular viewing and during binocular rivalry in order to determine changes in sensitivity brought about by perceptual suppression. We chose this experimental strategy because we felt it would maximize the amount of data generated in short periods of single-unit isolation and that it would be the most sensitive procedure for revealing small effects. An illustration of the experimental procedure is shown in Figures 7A,B. For each cell, we recorded spiking responses to a randomly-modulating (randomly transitioning between 0 and 180 degree phase) grating; trial conditions were randomly interleaved, either displaying a purely monocular modulating grating or a modulating grating presented simultaneously with a dichoptic suppressor [either an annulus (shown) or a high-contrast orthogonal grating]. The same random-noise sequence was used for both stimulation conditions in all cells; only complete runs through the entire stimulus sequence in both trial conditions were considered in our analysis.

As in the experiment with static gratings, we first partitioned the sample of recorded cells based on exhibited response patterns. Figure 7C shows our separation procedure: our sample of temporal kernels were first transformed to step responses and then decomposed into principal components. The normalized step responses were projected onto the first two principal components and clustered using the k-means algorithm seeded with two centroids. This procedure resulted in two stable clusters, one exhibiting transient response profiles and one exhibiting sustained response profiles.

The use of binocular rivalry suppression in this experiment is indirect; that is, we do not explicitly compare responses during epochs when a probe is reported to be suppressed or dominant, but rather measure the sensitivity of cells when a probe is presented alone (known to be dominant) or during periods of binocular rivalry when the probe is assumed to be perceptually suppressed for a significant proportion of the trial. To understand quantitatively what proportion of the time the probe is perceptually suppressed, we used three human observers to characterize the rivalry dynamics of the dichoptic stimulus we used in this experiment. Observers viewed the stimuli (using the same noise sequence and trial structure as the monkey experiment) through a mirror stereoscope and were instructed to hold down a button when the probe was visible. The total proportion of time through the entire sequence that the probes were reported visible is plotted in Figure 8A. The proportions are averaged over six full presentations of the noise sequence, using either the annulus or grating as a suppressor. The proportion of time the probes were reported visible was 0.4 (S.D. = 0.09) for the annulus suppressor and 0.39 (S.D. = 0.05) for the high-contrast grating suppressor. To avoid using an ambiguous perceptual reporting procedure with monkeys, we verified that the probe-annulus configuration elicited psychophysical suppression in Monkey B using a contrast change-detection procedure (see Methods). Averaged over all sessions, with stimulus eccentricities varying from 3 to 8 degrees, the dichoptic annulus elicited an increase in contrast change-detection threshold of approximately 65% (Figure 8B).

**Figure 9:**
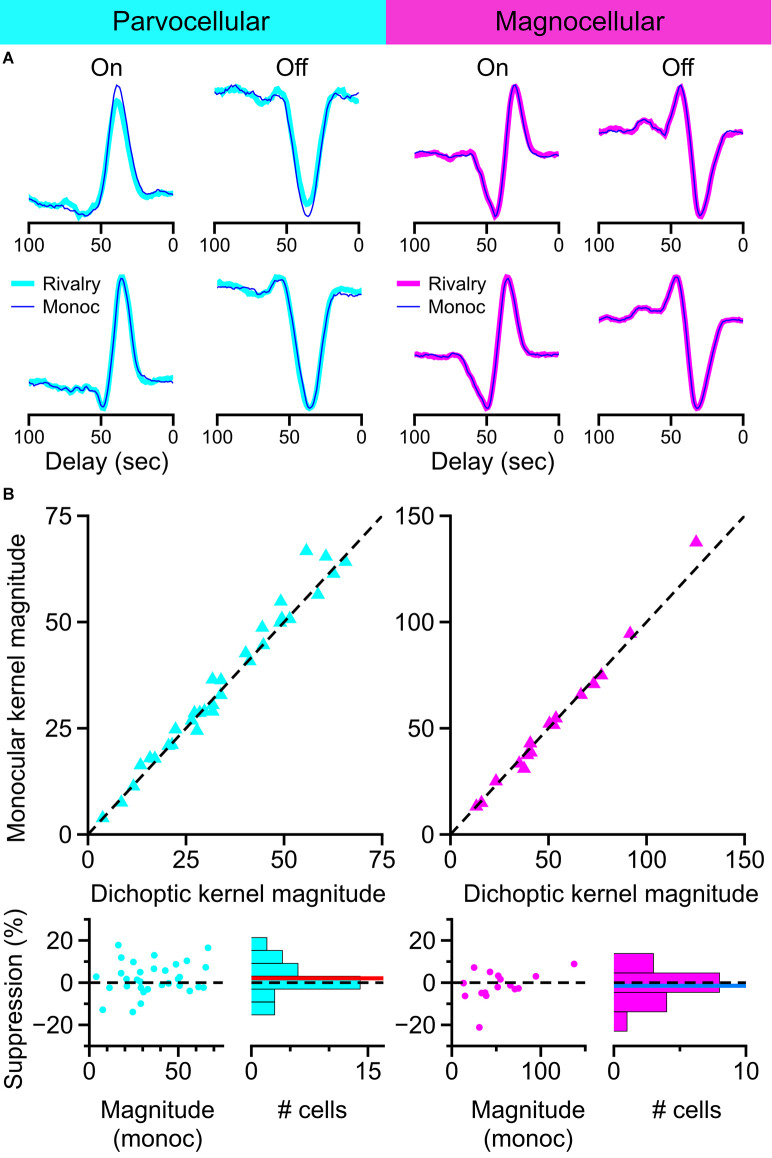
Influence of binocular rivalry on the magnitude of temporal kernels recovered from LGN cells. **(A)** Example temporal kernels recovered during monocular viewing (thin traces) and during binocular rivalry (thick traces) in parvocellular cells and magnocellular cells. **(B)** Comparison of kernel magnitudes obtained during monocular viewing and during binocular rivalry. Upper row: scatterplots of the magnitude of kernels recovered during monocular viewing plotted against the magnitude of kernels recovered during binocular rivalry. Lower row: suppression percentage for each cell is plotted against the cell’s monocular kernel magnitude; histogram of suppression percentage for all cells shown at right. Dashed line at 0 for reference, red line, and blue line represent mean suppression.

Representative examples of kernels recovered with this method are shown in Figure 9A. Examples of On-cells and Off-cells from the parvocellular cell cluster and the magnocellular cell cluster are shown, with kernels recovered during monocular viewing (thin lines) overlaid on kernels recovered during dichoptic viewing (thick lines). The magnitude of kernels recovered during monocular stimulation is plotted against the magnitude of kernels recovered during binocular rivalry in Figure 9B. After controlling for eye position differences between conditions, there was no suppressive influence of rivalry in the parvocellular cells (*t*-test, *p* = 0.24) or the magnocellular cells (*t*-test, *p* = 0.74); we did not observe a difference in rivalry suppression between On cells and Off cells (Welch’s *t*-test; parvocellular cells *p* = 0.42; magnocellular cells *p* = 0.10). These data are plotted and histogrammed as percent suppression (percent reduction of monocular kernel magnitude) in the bottom row of Figure 9B.

## Discussion

The goals of this study were threefold: (1) to determine the characteristics of surround suppression in the parallel streams (On-center, Off-center, magnocellular, and parvocellular) of the LGN; (2) to determine the extent to which non-dominant eye stimulation influences the spiking activity of LGN cells, and (3) to determine the magnitude of the effect of perceptual suppression on the sensitivity of LGN cells. Our data allowed us to characterize the timing and strength of both monocular surround suppression and non-dominant eye suppression, potentially enabling us to evaluate the possibility that they would be affected through the same circuits. We found evidence for two different components of monocular surround suppression—an early-stage suppression exhibited by all magnocellular cells, likely inherited from the retina (Alitto and Usrey, 2008), and a late-stage suppression exhibited only by Off cells in both the parvocellular and magnocellular pathways. Our results demonstrated that there is no detectable influence of non-dominant eye stimulation on the firing properties of parvocellular or magnocellular cells in the macaque LGN. Likewise, we found no evidence for rivalry-related suppression in our sample of LGN cells. Thus, under our experimental conditions, binocular interactions do not occur in the magnocellular or parvocellular layers of the primate LGN. This is in contrast to the response properties of the koniocellular cell layers; results from Cheong et al. (2013) and Belluccini et al. (2019) have demonstrated robust binocular summation by single neurons in the koniocellular cell layers. It is interesting that this phylogenetically older pathway may perform computations that contribute to binocular combination, whereas the more recent P and M pathways seem to remain more strongly segregated in eye channels prior to synapsing on neurons of the visual cortex.

In our experiment using brief presentations of static gratings, we observed two distinct patterns of surround suppression in LGN cells. In our sample of magnocellular cells, a powerful early-stage monocular surround suppression was present that is likely inherited from the retina (Alitto and Usrey, 2008). In our sample of Off cells from both the parvocellular and magnocellular groups, there was also a late-stage monocular surround suppression component that exhibited a delayed onset, with a latency of 42.3 ms from stimulus onset in the parvocellular cells, lagging the excitatory response latency in these cells by 15 ms. Briggs and Usrey (2007) found that the average response latency of V1 neurons providing corticogeniculate feedback to the LGN was 52 ms, with the shortest latency they observed in their sample being 37 ms. The slow onset of late-stage monocular surround suppression in Off cells may therefore be consistent with the involvement of cortical feedback, as suggested by Jones et al. (2012).

Previous reports of binocular interaction in the primate LGN have been heterogeneous. Both multi-unit (Schroeder et al., 1990) and single-unit (Marrocco and McClurkin, 1979; Rodieck and Dreher, 1979; Dougherty et al., 2021) studies have described binocular interactions in the primate LGN, though as a mixture of excitatory and inhibitory effects. Potential circuitry for such effects exists, through monosynaptic or disynaptic inhibition between LGN layers, disynaptic inhibition from the thalamic reticular nucleus, and feedback from the cortex. Further, studies in carnivores have suggested that binocular interaction at the level of the LGN is predominantly suppressive and likely the result of subcortical processing (Sanderson et al., 1971; Murphy and Sillito, 1989; Tumosa et al., 1989; Tong et al., 1992). While we did see wide variation in our experimental measurement of non-dominant eye effects, our results suggest that the net influence of non-dominant eye stimulation in the monkey LGN is negligible. It is worth noting that the range of contrasts predominantly used in our study is similar to the high-contrast and medium-contrast conditions of Dougherty et al. (2021), in which little or no binocular modulation was observed.

To characterize the influence of perceptual suppression on spiking activity in the LGN, we used a reverse-correlation procedure to estimate the magnitude of the temporal receptive fields of LGN cells during binocular rivalry and monocular viewing. Because of negative results reported by previous groups (Lehky and Maunsell, 1996; Wilke et al., 2009), we chose this stimulus configuration based on its potential sensitivity, assuming that any effects, if present, would be difficult to detect. We found no influence of rivalry suppression on the magnitude of temporal kernels recovered from parvocellular cells or magnocellular cells.

Despite our expectation of a small effect of rivalry on neuronal activity, we note that Wunderlich et al. (2005) and Haynes et al. (2005) have independently reported extremely large modulations of the BOLD response in the LGN that correlate with eye-specific perceptual oscillations during binocular rivalry. The issue of widely diverging measurements of perceptual modulation between single-units and fMRI has been treated rigorously by Maier et al. (2008), where they measured the modulation of BOLD activity, LFP, and spiking responses by a single perceptual suppression procedure in V1 of alert monkeys, and convincingly demonstrated that the wide divergence of modulation of these signals does not depend on task (or species) variables. While it is still tempting to hypothesize that not requiring perceptual reporting could be another source of divergence between single-unit studies and fMRI, the close agreement of the magnitude of perceptual effects found in unit measurements in V1 made concurrently with perceptual reporting (Leopold and Logothetis, 1996) and those not made concurrently with perceptual reporting (Keliris et al., 2010), as well as similar magnitudes of measured suppression in V1 of alert and anesthetized animals (Bahmani et al., 2014), suggest that this is not the case.

In closing, this study provided a rigorous analysis of the involvement of the LGN in the contextual modulation of feedforward inputs to the cortex, and it identified clear differences in the magnitude and time course of suppression in the parallel streams of visual processing within the LGN. The results show that at the earliest stages of the visual system, monocular channels in the parvocellular and magnocellular streams remain strictly segregated. Results further demonstrate that surround suppression measured in the M layers of the LGN exhibits an early component in which suppression is similar for On and Off-center cells, and a late-stage component which is carried predominantly by the Off channel; surround suppression measured in the P layers only exhibited a late-stage component that was carried almost entirely by the Off channels. Together, these results extend our understanding of the hierarchy of binocular vision and visual suppression in the primate and provide a clearer view of the modularity of the early visual system and the manner in which specific tasks are performed within its distinct pathways.

## Data Availability Statement

The raw data supporting the conclusions of this article will be made available by the authors, without undue reservation.

## Ethics Statement

The studies involving human participants were reviewed and approved by Institutional Review Board (IRB) Administration, University of California, Davis. The patients/participants provided their written informed consent to participate in this study. The animal study was reviewed and approved by Institutional Animal Care and Use Committee (IACUC) University of California, Davis.

## Author Contributions

BL and WU conceived the project, wrote, and edited the manuscript. BL collected and analyzed data. All authors contributed to the article and approved the submitted version.

## Conflict of Interest

The authors declare that the research was conducted in the absence of any commercial or financial relationships that could be construed as a potential conflict of interest.

## Publisher’s Note

All claims expressed in this article are solely those of the authors and do not necessarily represent those of their affiliated organizations, or those of the publisher, the editors and the reviewers. Any product that may be evaluated in this article, or claim that may be made by its manufacturer, is not guaranteed or endorsed by the publisher.
